# Do guidelines influence breathlessness management in advanced lung diseases? A multinational survey of respiratory medicine and palliative care physicians

**DOI:** 10.1186/s12890-022-01835-0

**Published:** 2022-01-19

**Authors:** Małgorzata Krajnik, Nilay Hepgul, Andrew Wilcock, Ewa Jassem, Tomasz Bandurski, Silvia Tanzi, Steffen T. Simon, Irene J. Higginson, Caroline J. Jolley, the BETTER-B research consortium, Agnieszka Arendt-Nowakowska, Sabrina Bajwah, Claudia Bausewein, Jeremias Bazata, Charlotte Bolton, Candida Bonelli, Richard Brindle, Sarah Brown, Massimo Costantini, David Currow, Claire Dimbleby, Olivia Dix, Peter Doran, Yvonne Eisenmann, Alasdair Fellows, Malgorzata Fopka-Kowalczyk, Giovanni Gambassi, Nilay Hepgul, Irene Higginson, Amy Holton, Rabia Hussain, Piotr Janowiak, Ewa Jassem, Gisli Jenkins, Jingjing Jiang, Miriam Johnson, Caroline Jolley, Eszter Katona, Emer Kelly, Mateusz Kirjak, Malgorzata Krajnik, Matthew Maddocks, Anna Malara, Domenico Merlo, Hinna Mir, Brenda Molloy, Geraldine Murden, Charles Normand, Margaret Ogden, Adejoke Oluyase, Sabina Panfilak, Pippa Powell, Anne Pralong, Jackie Pullen, Faye Regan, Karen Ryan, Steffen Simon, Samantha Smith, Silvia Tanzi, Valerie Vaccaro, Raymond Voltz, Fiona Walker, Andrew Wilcock

**Affiliations:** 1 Department of Palliative Care, Collegium Medicum in Bydgoszcz, Nicolaus Copernicus University in Toruń, Skłodowskiej-Curie 9, 85-094 Bydgoszcz, Poland; 2 Cicely Saunders Institute of Palliative Care, Policy and Rehabilitation, King’s College London, London, UK; 3 Palliative Medicine, Hayward House Specialist Palliative Care Unit, Nottingham University Hospitals NHS Trust, University of Nottingham, Nottingham, UK; 4 Department of Pneumonology, Medical University of Gdańsk, Gdańsk, Poland; 5 Department of Radiology, Informatics and Statistics, Medical University of Gdańsk, Gdańsk, Poland; 6 Palliative Care Unit, Azienda USL-IRCCS Reggio Emilia, Reggio Emilia, Italy; 7 Department of Palliative Medicine and Center for Integrated Oncology Aachen Bonn Cologne Duesseldorf (CIO ABCD), Faculty of Medicine and University Hospital, University of Cologne, Cologne, Germany; 8 Centre for Human & Applied Physiological Sciences, School of Basic & Medical Biosciences, Faculty of Life Sciences & Medicine, King’s College London, Shepherd’s House, Rm 4.4, Guy’s Campus, London, SE1 1UL UK

**Keywords:** Dyspnea, Breathlessness, Surveys and Questionnaires, Pulmonary disease, Chronic obstructive, Lung diseases, Interstitial, Lung neoplasms, Palliative care

## Abstract

**Background:**

Respiratory medicine (RM) and palliative care (PC) physicians’ management of chronic breathlessness in advanced chronic obstructive pulmonary disease (COPD), fibrotic interstitial lung disease (fILD) and lung cancer (LC), and the influence of practice guidelines was explored via an online survey.

**Methods:**

A voluntary, online survey was distributed to RM and PC physicians via society newsletter mailing lists.

**Results:**

450 evaluable questionnaires (348 (77%) RM and 102 (23%) PC) were analysed. Significantly more PC physicians indicated routine use (often/always) of opioids across conditions (COPD: 92% vs. 39%, fILD: 83% vs. 36%, LC: 95% vs. 76%; all *p* < 0.001) and significantly more PC physicians indicated routine use of benzodiazepines for COPD (33% vs. 10%) and fILD (25% vs. 12%) (both *p* < 0.001). Significantly more RM physicians reported routine use of a breathlessness score (62% vs. 13%, *p* < 0.001) and prioritised exercise training/rehabilitation for COPD (49% vs. 7%) and fILD (30% vs. 18%) (both *p* < 0.001). Overall, 40% of all respondents reported reading non-cancer palliative care guidelines (either carefully or looked at them briefly). Respondents who reported reading these guidelines were more likely to: routinely use a breathlessness score (*χ*^2^ = 13.8; *p* < 0.001), use opioids (*χ*^2^ = 12.58, *p* < 0.001) and refer to pulmonary rehabilitation (*χ*^2^ = 6.41, *p* = 0.011) in COPD; use antidepressants (*χ*^2^ = 6.25; *p* = 0.044) and refer to PC (*χ*^2^ = 5.83; *p* = 0.016) in fILD; and use a handheld fan in COPD (*χ*^2^ = 8.75, *p* = 0.003), fILD (*χ*^2^ = 4.85, *p* = 0.028) and LC (*χ*^2^ = 5.63; *p* = 0.018).

**Conclusions:**

These findings suggest a need for improved dissemination and uptake of jointly developed breathlessness management guidelines in order to encourage appropriate use of existing, evidence-based therapies. The lack of opioid use by RM, and continued benzodiazepine use in PC, suggest that a wider range of acceptable therapies need to be developed and trialled.

**Supplementary Information:**

The online version contains supplementary material available at 10.1186/s12890-022-01835-0.

## Background

Breathlessness is a distressing, highly prevalent symptom of advanced chronic respiratory diseases and lung cancer (LC) [1–4]. It is associated with social isolation [5], high healthcare costs [6, 7] and poor prognosis [8]. Chronic or refractory breathlessness is defined as disabling breathlessness which persists despite optimal disease management [9]. It may be episodic or persistent, and usually becomes increasingly severe with disease progression and at end of life [10]. Management options include non-pharmacological interventions e.g. exercise/rehabilitation, use of a handheld fan, breathing control techniques and walking aids [11]. Pharmacological treatment options are limited to moderate evidence in support of opioids [12, 13]. Thus, breathlessness often remains under-recognised and undertreated.

Recent multinational and national surveys have highlighted considerable variation in treatment approaches for breathlessness between respiratory medicine (RM) and palliative care (PC) physicians [14], and in the approach to breathlessness management in malignant *versus* non-malignant disease [15]. Barriers to effective management include lack of clinician knowledge and experience [16–18]. Furthermore, a survey of 174 Polish Respiratory Society members suggested an inverse relationship between knowledge of PRS guidelines and treatments recommended for people with chronic obstructive pulmonary disease (COPD) [15]. Thus, the aim of this survey was to describe and compare the management practices of RM and PC physicians across Europe for breathlessness in chronic lung diseases and to explore the relationship between the knowledge of guidelines and clinical practice.

## Methods

### Study design and participants

This survey was conducted as part of the BETTER-B research programme on breathlessness in advanced diseases. An anonymous, voluntary online survey was designed, in English, for distribution to physicians working in RM and PC across Europe. Survey design was informed by previous surveys [14, 15, 17, 18] and current literature [11, 12, 19]. Three case vignettes were developed: one patient with advanced COPD, one with progressive fibrotic interstitial lung disease (fILD) (a case of advanced idiopathic pulmonary fibrosis (IPF)), and one patient with LC (see Additional file 1). Each patient presented with mMRC scale 3–4 breathlessness (3 = Stops for breath after walking 100 yards, or after a few minutes on level ground; 4 = Too breathless to leave the house, or breathless when dressing/undressing) [20] despite optimal management of the underlying disease. Current anxiety or depression was not indicated in any of the three vignettes. The survey focused on: respondent demographics; awareness and knowledge of local, national or international guidelines/recommendations on palliative care for non-malignant lung diseases; use of a breathlessness score in clinical practice; non-pharmacological management strategies; pharmacological management strategies; and attitudes towards referral to PC. Respondents were asked to consider how they would manage each case vignette, or similar patients, by rating management/treatment options.

The survey was piloted on 20 international expert RM and PC physicians (data not included in the final analyses). Additionally, 10 in-depth interviews were performed among physicians from Germany, Italy, Poland and the UK to minimise measurement error and ensure user acceptability, face validity and comprehensiveness. The survey was launched on 23/04/2019 and closed on 06/08/2019. Survey links were disseminated via newsletter mailing lists to members of the European Respiratory Society (ERS), the European Association for Palliative Care (EAPC) and the British Thoracic Society (BTS), and as a news item feature on the Palliative Care Formulary (PCF) website. Society members were further encouraged to participate through social media posts, blogs, and dissemination among linked national societies and conferences. Approval was granted by the King’s College London (UK) Research Ethics Committee (MRA-18/19-11108). Physicians were informed that by completing the survey, they provided informed consent for use of their anonymised data.

### Analysis

Analyses were performed using IBM SPSS Statistics version 25.0 and STATISTICA StatSoft version 12.0. Categories were collapsed or dichotomized for some analyses. Pearson’s chi-square test, Yates’ correction, or Fisher’s exact test were used to compare frequencies and proportions between RM and PC physicians. As there was no significant difference between RM and PC physicians in their knowledge of guidelines on PC for non-malignant lung diseases, the impact of guideline knowledge on clinical practice was analysed as one sample. Answers were dichotomised as: 1—yes, I know of them and have read them carefully and yes, I know of them but have only looked at them briefly versus 2—the three other response options (see Additional file 1). Logistic regressions were carried out to evaluate which respondent characteristics (from Table 1) were independently associated with dichotomised knowledge of guidelines. Variables which presented statistical significance in the univariate analyses (setting of practice, number of COPD patients seen, number of fILD patients seen and number of LC patients seen) were subsequently entered into a multivariate logistic regression model. Significant associations are presented as odds ratios (OR), their respective 95% confidence intervals (CI) and significance levels. Further, knowledge of guidelines was transformed into a 5-point Likert scale: 1—I know that no such guidelines/recommendations exist; 2—I’m not sure if such guidelines/recommendations exist or not; 3—Yes, I know of them but have not read them; 4—Yes, I know of them but have only looked at them briefly; and 5—Yes, I know of them and have read them carefully. Mann–Whitney-Wilcoxon test were used to compare groups for ordinal variables. Kruskal–Wallis test was used to assess differences in ordinal data among three or more independently sampled groups with post hoc Dunn’s multiple comparison.

**Table 1:** Respondent characteristics

	RM (n = 348)	PC (n = 102)	Specialties compared (*χ*^2^) *p* value
*Age*			
25–35	55 (16%)	19 (19%)	*p* = 0.181
36–45	123 (35%)	33 (32%)	
46–55	86 (25%)	33 (32%)	
> 56	84 (24%)	16 (16%)	
*Grade*			
Consultant/specialist	312 (90%)	81 (79%)	*p* = 0.006
Doctor in specialist training	36 (10%)	21 (21%)	
*Years in specialty*			
< 5	41 (12%)	21 (21%)	*p* = 0.001
6–10	75 (22%)	23 (23%)	
11–20	101 (29%)	40 (39%)	
> 21	131 (38%)	18 (18%)	
*Settings of practice*			
Hospital inpatient	295 (85%)	54 (53%)	*p* < 0.001
Outpatient	218 (63%)	37 (36%)	*p* < 0.001
Home care	11 (3%)	39 (38%)	*p* < 0.001
Private practice	56 (16%)	4 (4%)	*p* = 0.001
Hospice/palliative care unit	4 (1%)	68 (67%)	*p* < 0.001
Other	8 (2%)	5 (5%)	*p* = 0.167
*No. of severe COPD patients seen/year*			
None	12 (3%)	7 (7%)	*p* < 0.001
1–10	87 (25%)	43 (42%)	
11–50	162 (47%)	43 (42%)	
51–100	57 (16%)	7 (7%)	
> 101	30 (9%)	2 (2%)	
*No. of severe fILD patients seen/year*			
None	24 (7%)	15 (15%)	*p* = 0.001
1–5	118 (34%)	48 (47%)	
6–10	97 (28%)	22 (22%)	
11–20	58 (17%)	12 (12%)	
> 20	51 (15%)	5 (5%)	
*No. of advanced LC patients seen/year*			
None	48 (14%)	–	*p* < 0.001
1–10	123 (35%)	11 (11%)	
11–50	131 (37%)	42 (41%)	
51–100	28 (8%)	33 (32%)	
> 101	18 (5%)	16 (16%)	

Percentages > or < 100% are due to rounding

COPD, Chronic Obstructive Pulmonary Disease; fILD, Fibrotic Interstitial Lung Disease; LC, Lung Cancer; PC, Palliative Care; RM, Respiratory Medicine

## Results

### Respondent characteristics

**Fig. 1:**
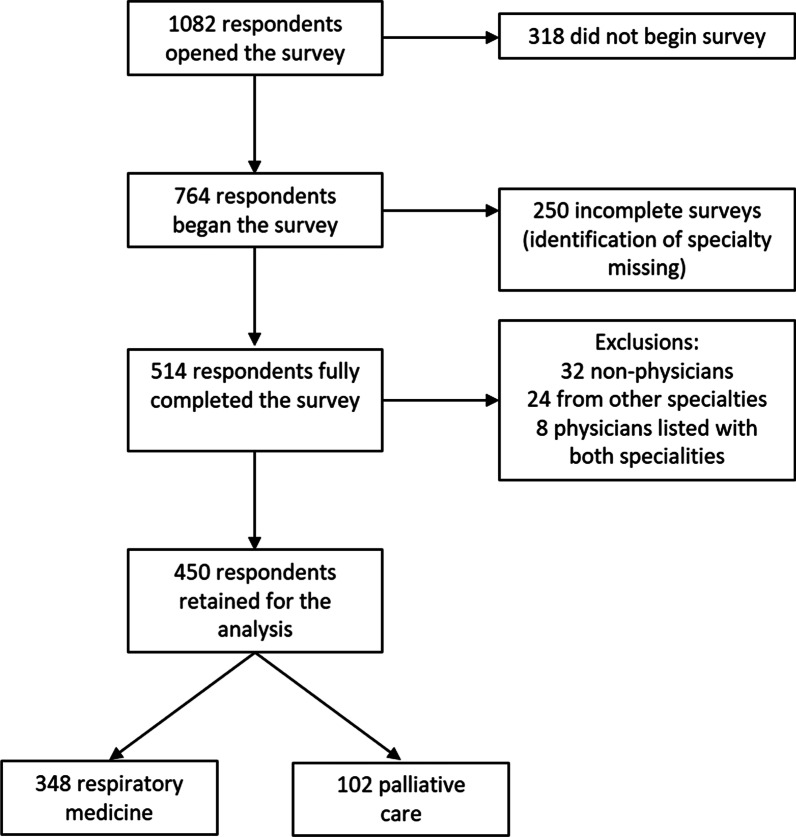
Flow of survey responses

The survey was opened by 1082 recipients, commenced by 764 respondents with 514 complete and 250 partial responses. Following exclusions, 450 responses were included in the final analyses with 348 (77%) RM and 102 (23%) PC physicians (Table 1 and Fig. 1). RM physicians practiced across 31 and PC across 13 European countries, with largest representation from the UK (18% and 36%, respectively). A further 59 (13%) responses were from non-European countries including India, USA and several South American countries. PC and RM physicians differed according to years in their specialty, settings in which they work, and numbers of patients seen.

### Non-pharmacological management of breathlessness

For chronic breathlessness in COPD and fILD, RM physicians most commonly recommended (“often or always”) physical activity (COPD 71%, fILD 61%), pulmonary rehabilitation (COPD 67%, fILD 56%) and breathing techniques (COPD 58%, fILD 42%). By contrast, PC physicians favoured breathing techniques (COPD 73%, fILD 69%), body positioning (COPD 70%, fILD 68%) and the handheld fan (COPD 66%, fILD 64%).

In LC, PC physicians most commonly recommended body positioning, which was selected by significantly fewer RM physicians (72% vs. 32%, *p* < 0.001). The handheld fan was also more frequently selected by PC physicians with 57% of RM physicians reporting that they “never” recommend the handheld fan in LC. Half of physicians from both specialties reported only “rarely or sometimes” recommending physical activity (RM 52%, PC 54%, *p* = 0.453) and “never” recommending pulmonary rehabilitation (RM 49%, PC 55%, *p* = 0.602) for breathlessness in LC. Meditative and cognitive interventions were less commonly recommended (“often or always”) across all three cases by both PC and RM physicians (see Table 2).

**Table 2:** Choice of non-pharmacological treatment strategies, in response to the case vignettes, compared between respiratory medicine (RM) and palliative care (PC) physicians

	COPD			fILD			LC		
	RM (n = 336)	PC (n = 95)	Specialties compared (*χ*^2^) *p* value	RM (n = 324)	PC (n = 87)	Specialties compared (*χ*^2^) *p* value	RM (n = 301)	PC (n = 102)	Specialties compared (*χ*^2^) *p* value
*Pulmonary rehabilitation*									
Often or always	226 (67%)	27 (28%)	*p* < 0.001	181 (56%)	26 (30%)	*p* < 0.001	32 (11%)	10 (10%)	*p* = 0.602
Rarely or sometimes	101 (30%)	43 (45%)		125 (39%)	43 (49%)		121 (40%)	36 (35%)	
Never	9 (3%)	25 (26%)		18 (6%)	18 (1%)		148 (49%)	56 (55%)	
*Physical activity*									
Often or always	240 (71%)	33 (35%)	*p* < 0.001	199 (61%)	27 (31%)	*p* < 0.001	70 (23%)	18 (18%)	*p* = 0.453
Rarely or sometimes	87 (26%)	47 (50%)		111 (34%)	46 (53%)		157 (52%)	55 (54%)	
Never	9 (3%)	15 (16%)		14 (4%)	14 (16%)		74 (25%)	29 (28%)	
*Electric handheld fan*									
Often or always	61 (18%)	63 (66%)	*p* < 0.001	55 (17%)	56 (64%)	*p* < 0.001	59 (20%)	64 (63%)	*p* < 0.001
Rarely or sometimes	93 (28%)	20 (21%)		85 (26%)	18 (21%)		71 (24%)	22 (21%)	
Never	182 (54%)	12 (13%)		184 (57%)	13 (15%)		170 (57%)	16 (16%)	
*Breathing techniques*									
Often or always	195 (58%)	69 (73%)	*p* = 0.010	137 (42%)	60 (69%)	*p* < 0.001	85 (28%)	62 (61%)	*p* < 0.001
Rarely or sometimes	115 (34%)	17 (18%)		134 (41%)	18 (21%)		145 (48%)	27 (27%)	
Never	26 (8%)	9 (10%)		53 (16%)	9 (10%)		71 (24%)	13 (13%)	
*Respiratory muscle training*									
Often or always	153 (46%)	17 (18%)	*p* < 0.001	114 (35%)	23 (26%)	*p* = 0.004	35 (12%)	17 (17%)	*p* = 0.411
Rarely or sometimes	118 (35%)	42 (44%)		134 (41%)	28 (32%)		126 (42%)	39 (38%)	
Never	65 (19%)	36 (38%)		76 (24%)	36 (41%)		140 (47%)	46 (45%)	
*Body positioning to relieve breathlessness*									
Often or always	147 (44%)	66 (70%)	*p* < 0.001	101 (31%)	59 (68%)	*p* < 0.001	95 (32%)	73(72%)	*p* < 0.001
Rarely or sometimes	114 (34%)	24 (25%)		123 (38%)	23 (26%)		115 (38%)	22 (22%)	
Never	75 (22%)	5 (5%)		100 (31%)	5 (6%)		90 (30%)	7 (7%)	
*Walking aids*									
Often or always	149 (44%)	63 (66%)	*p* < 0.001	111 (34%)	54 (62%)	*p* < 0.001	102 (34%)	65 (64%)	*p* < 0.001
Rarely or sometimes	132 (39%)	26 (27%)		145 (45%)	26 (30%)		129 (43%)	29 (28%)	
Never	55 (16%)	6 (6%)		68 (21%)	7 (8%)		70 (23%)	8 (8%)	
*Meditative interventions*									
Often or always	34 (10%)	23 (24%)	*p* < 0.001	34 (11%)	27 (31%)	*p* < 0.001	44 (15%)	22 (22%)	*p* = 0.005
Rarely or sometimes	109 (32%)	44 (46%)		108 (33%)	34 (39%)		104 (35%)	47 (46%)	
Never	193 (57%)	28 (30%)		182 (56%)	26 (30%)		153 (51%)	33 (32%)	
*Cognitive/emotional interventions*									
Often or always	48 (14%)	28 (30%)	*p* = 0.001	49 (15%)	29 (33%)	*p* = 0.001	75 (25%)	31 (30%)	*p* = 0.195
Rarely or sometimes	162 (48%)	45 (47%)		152 (47%)	34 (39%)		124 (41%)	46 (45%)	
Never	126 (38%)	22 (23%)		123 (38%)	24 (28%)		102 (34%)	25 (25%)	

Percentages > or < 100% are due to rounding

COPD, Chronic Obstructive Pulmonary Disease; fILD, Fibrotic Interstitial Lung Disease; LC, Lung Cancer; PC, Palliative Care; RM, Respiratory Medicine

### Pharmacological management of breathlessness

Opioids were more commonly recommended “often or always” than benzodiazepines or antidepressants by both RM and PC physicians for all three cases (Table 3).

**Table 3:** Choice of pharmacological treatment strategies, in response to the case vignettes, compared between respiratory medicine (RM) and palliative care (PC) physicians

	COPD			fILD			LC		
	RM (n = 336)	PC (n = 95)	Specialties compared (*χ*^2^) *p* value	RM (n = 324)	PC (n = 87)	Specialties compared (*χ*^2^) *p* value	RM (n = 300)	PC (n = 102)	Specialties compared (*χ*^2^) *p* value
*Opioids*									
Often or always	132 (39%)	87 (92%)	*p* < 0.001	117 (36%)	72 (83%)	*p* < 0.001	227 (76%)	97 (95%)	*p* < 0.001
Rarely or sometimes	150 (45%)	7 (7%)		148 (46%)	14 (16%)		59 (20%)	5 (5%)	
Never	54 (16%)	1 (1%)		59 (18%)	1 (1%)		14 (5%)	–	
*Benzodiazepines*									
Often or always	34 (10%)	31 (33%)	*p* < 0.001	40 (12%)	22 (25%)	*p* < 0.001	108 (36%)	47 (46%)	*p* = 0.001
Rarely or sometimes	194 (58%)	60 (63%)		181 (56%)	58 (67%)		142 (47%)	52 (51%)	
Never	108 (32%)	4 (4%)		103 (32%)	7 (8%)		50 (17%)	3 (3%)	
*Antidepressants*									
Often or always	62 (19%)	10 (11%)	*p* = 0.010	39 (12%)	11 (13%)	*p* = 0.298	63 (21%)	15 (15%)	*p* = 0.379
Rarely or sometimes	201 (60%)	73 (77%)		175 (54%)	54 (62%)		173 (58%)	63 (62%)	
Never	73 (22%)	12 (13%)		110 (34%)	22 (25%)		64 (21%)	24 (24%)	

Percentages > or < 100% are due to rounding

COPD, Chronic Obstructive Pulmonary Disease; fILD, Fibrotic Interstitial Lung Disease; LC, Lung Cancer; PC, Palliative Care; RM, Respiratory Medicine

However, opioids were selected “often or always” by significantly more PC in both COPD (92% vs. 39%, *p* < 0.001) and fILD (83% vs. 36%, *p* < 0.001). This was also observed for LC, although the difference was smaller (95% vs. 76%, *p* < 0.001). Conversely, larger proportions of RM physicians stated they would never initiate opioids in severe COPD (16% vs. 1%) or fILD (18% vs. 1%). The three commonest reasons selected by RM physicians for not, or only rarely, initiating opioids for patients with severe COPD or fILD were: risk of respiratory depression (COPD 20%, fILD 14%), risk of unpleasant side-effects (COPD 15%, fILD 12%) and insufficient knowledge or experience in prescribing opioids in these patients (15% for both COPD and fILD). Benzodiazepines were less frequently selected by RM physicians across cases. Specifically, one-third (32%) stated that they would “never” select benzodiazepines for COPD and fILD. Conversely, 33% and 25% of PC physicians would “often or always” recommend benzodiazepines for COPD and fILD respectively (compared to RM: 10% and 12%, *p* < 0.001). One fifth or fewer physicians from both specialties would routinely recommend antidepressants in the management of breathlessness, across all three cases. Among those who would consider antidepressants for COPD, 30% of RM and 47% of PC physicians, stated they would not use antidepressants solely for the management of breathlessness. When used, selective serotonin reuptake inhibitors (SSRIs) were more commonly selected than other classes of antidepressants by more than half of RM physicians (Table 4). PC physicians favoured both SSRIs and noradrenergic and specific serotonergic antidepressant (NaSSAs), such as mirtazapine.

**Table 4:** Choice of pharmacological treatments for chronic breathlessness, in response to the case vignettes, compared between respiratory medicine (RM) and palliative care (PC) physicians

	COPD			fILD			LC		
	RM (n = 207–263)*	PC (n = 83–93)*	Specialties compared (*χ*^2^) *p* value	RM (n = 193–214)*	PC (n = 65–83)*	Specialties compared (*χ*^2^) *p* value	RM (n = 236–265)*	PC (n = 78–99)*	Specialties compared (*χ*^2^) *p* value
*Opioids*									
Oral dihydrocodeine regularly	6 (3%)	–	*p* = 0.042	8 (4%)	–	*p* = 0.402	11 (4%)	–	*p* = 0.003
Short-acting oral morphine PRN	79 (38%)	35 (38%)		81 (42%)	44 (53%)		90 (34%)	30 (30%)	
Short-acting oral morphine regularly	45 (22%)	31 (33%)		36 (19%)	17 (21%)		54 (20%)	32 (32%)	
Long-acting oral morphine regularly	57 (28%)	20 (22%)		50 (26%)	17 (21%)		84 (32%)	25 (25%)	
Subcutaneous morphine injection PRN	10 (5%)	–		7 (4%)	1 (1%)		8 (3%)	–	
Subcutaneous morphine injection regularly or continuous	1 (1%)	2 (2%)		2 (1%)	1 (1%)		8 (3%)	9 (9%)	
Other short-acting PRN	9 (5%)	5 (5%)		9 (5%)	3 (3%)		10 (4%)	3 (3%)	
*Benzodiazepines*									
Long-acting orally PRN	21 (10%)	4 (5%)	*p* < 0.001	20 (9%)	2 (3%)	*p* < 0.001	26 (11%)	1 (1%)	*p* < 0.001
Long-acting orally regularly	21 (10%)	1 (1%)		23 (11%)	1 (1%)		37 (15%)	6 (6%)	
Intermediate-acting orally PRN	98 (44%)	60 (69%)		99 (47%)	59 (78%)		80 (33%)	60 (61%)	
Intermediate-acting orally regularly	38 (17%)	7 (8%)		46 (22%)	7 (9%)		69 (28%)	13 (13%)	
Short-acting subcutaneously PRN	40 (18%)	9 (10%)		21 (10%)	6 (8%)		28 (11%)	11 (11%)	
Short-acting subcutaneously regularly	2 (1%)	2 (2%)		4 (2%)	1 (1%)		6 (2%)	7 (7%)	
Other	1 (1%)	4 (5%)		–	–		–		
*Antidepressants*									
SSRI	135 (51%)	17 (21%)	*p* < 0.001	118 (55%)	20 (31%)	*p* < 0.001	125 (53%)	18 (23%)	*p* < 0.001
NaSSA	22 (8%)	22 (27%)		22 (10%)	21 (32%)		23 (10%)	27 (35%)	
Tricyclic	21 (8%)	4 (5%)		16 (8%)	1 (2%)		19 (8%)	5 (6%)	
SNRI	4 (2%)	1 (1%)		4 (2%)	1 (2%)		11 (5%)	2 (3%)	
Other	1 (1%)	–		5 (2%)	–		3 (1%)	–	
Not used for breathlessness only	80 (30%)	39 (47%)		49 (23%)	22 (34%)		55 (23%)	26 (33%)	

Percentages > or < 100% are due to rounding

COPD, Chronic Obstructive Pulmonary Disease; fILD, Fibrotic Interstitial Lung Disease; LC, Lung Cancer; PC, Palliative Care; RM, Respiratory Medicine. *Varied “n” based on differing rates of prescribing for each category of pharmacological treatment

### Prioritised treatment and referrals to palliative care

RM physicians most commonly prioritised exercise/rehabilitation for COPD (49%), and drug treatment for LC (58%) (Table 5).

**Table 5:** Treatment priorities, in response to the case vignettes, compared between respiratory medicine (RM) and palliative care (PC) physicians

	COPD			fILD			LC		
	RM (n = 336)	PC (n = 95)	Specialties compared (*χ*^2^) *p* value	RM (n = 324)	PC (n = 87)	Specialties compared (*χ*^2^) *p* value	RM (n = 300)	PC (n = 102)	Specialties compared (*χ*^2^) *p* value
Drug treatment for breathlessness	70 (21%)	52 (55%)	*p* < 0.001	78 (24%)	35 (40%)	*p* < 0.001	174 (58%)	76 (75%)	*p* = 0.001
Re-assess oxygen prescription	29 (9%)	2 (2%)		79 (24%)	3 (3%)		23 (8%)	1 (1%)	
Non-pharmacological, non-exercise intervention	28 (8%)	25 (26%)		28 (9%)	24 (28%)		29 (10%)	15 (15%)	
Exercise training / rehabilitation	166 (49%)	7 (7%)		96 (30%)	16 (18%)		10 (3%)	1 (1%)	
Psychological assessment to explore co-existing anxiety and/or depression	31 (9%)	5 (5%)		30 (9%)	6 (7%)		58 (19%)	7 (7%)	
Other	12 (4%)	4 (4%)		13 (4%)	3 (3%)		6 (2%)	2 (2%)	

Percentages > or < 100% are due to rounding

COPD, Chronic Obstructive Pulmonary Disease; fILD, Fibrotic Interstitial Lung Disease; LC, Lung Cancer; PC, Palliative Care; RM, Respiratory Medicine

For fILD, their prioritised treatment was more evenly balanced between drug treatment (24%), exercise/rehabilitation (30%), and re-assessment of oxygen prescription (24%). PC physicians prioritised drug treatment regardless of diagnosis but especially for LC (75%). Across all three cases, most RM physicians stated that they would refer such patients to PC to provide ongoing palliation of breathlessness and other symptoms or for advice about palliation of breathlessness (COPD 73%, fILD 71%, LC 93%).

### Knowledge of PC practice guidelines and use of a breathlessness score

Only 15% of RM, and 17% of PC physicians reported that they knew of and had read carefully any local, national or international guidelines or recommendations on PC for non-malignant respiratory diseases (Table 6). Where examples of guidelines were given, these were predominantly national guidelines (e.g. British Thoracic Society Guidelines for Management of COPD, Spanish COPD Guidelines (GesEPOC), Danish respiratory society position paper on palliative care in patients with chronic progressive non-malignant lung diseases). Almost half of both specialties responded that no such guidelines/recommendations existed, or that they were unsure whether guidelines existed. Over two-thirds (62%) of RM physicians reported routinely using a breathlessness score in clinical practice (often or always) compared to 13% of PC physicians.

**Table 6:** Awareness of guidelines and use of a breathlessness score compared between respiratory medicine (RM) and palliative care (PC) physicians

	RM (n = 348)	PC (n = 102)	Specialties compared (*χ*^2^) *p* value
*Awareness of guidelines*			
Yes, I know of them and have read them carefully	53 (15%)	17 (17%)	*p* = 0.619
Yes, I know of them but have only looked at them briefly	86 (25%)	23 (23%)	
Yes, I know of them but have not read them	43 (12%)	12 (12%)	
I know that no such guidelines/recommendations exist	36 (10%)	6 (6%)	
I’m not sure if such guidelines/recommendations exist or not	130 (37%)	44 (43%)	
*Use of a breathlessness score*			
Yes, I routinely use a breathlessness score	215 (62%)	13 (13%)	*p* < 0.001
Yes, I sometimes use a breathlessness score	102 (29%)	26 (26%)	
No, I never use a breathlessness score	25 (7.0%)	57 (56%)	
No, I don’t know any breathlessness scores	6 (2%)	6 (6.0%)	

Percentages > or < 100% are due to rounding

PC, Palliative Care; RM, Respiratory Medicine

### Relationship between knowledge of guidelines and clinical practice

**Fig. 2:**
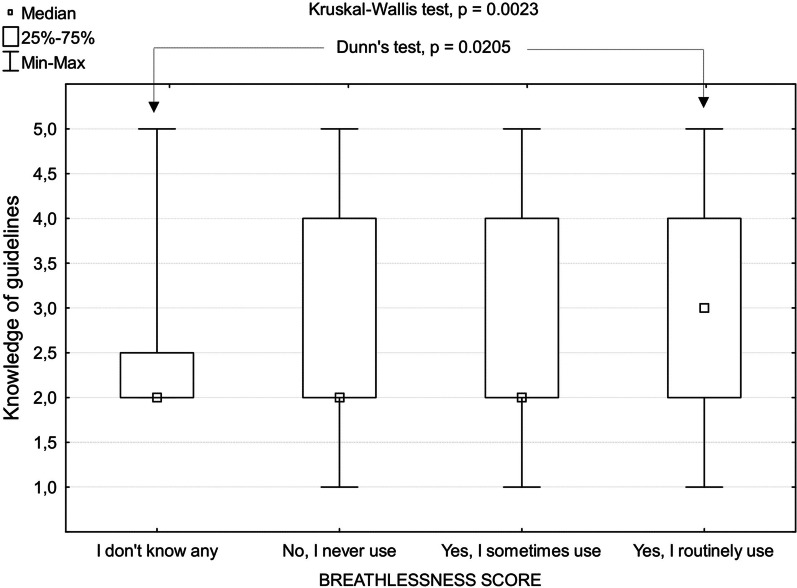
Relationship between the knowledge of guidelines/recommendations on palliative care for non-malignant lung diseases and the routine use of a breathlessness score in clinical practice. Legend: Knowledge of guidelines was evaluated by a 5-point Likert scale: 1 - I know that no such guidelines/recommendations exist; 2 - I’m not sure if such guidelines/recommendations exist or not; 3 - Yes, I know of them but have not read them; 4 - Yes, I know of them but have only looked at them briefly; 5 - Yes, I know of them and have read them carefully. Kruskal-Wallis test was implemented to assess the difference in ordinal data among all independently sampled groups, with subsequent post-hoc test (Dunn’s test) for multiple comparison

Respondents who treated a higher number of COPD patients a year reported greater knowledge of clinical guidelines for PC in non-malignant lung disease (read them either carefully or looked at them briefly) (OR 1.45; [CI 1.18–1.79]; *p* < 0.001). Physicians who read guidelines either carefully or looked at them briefly more often used a breathlessness score routinely in clinical practice (*χ*^2^ = 13.8; *p* < 0.001) and more often reported routine use of opioids to relieve chronic breathlessness in severe COPD (*χ*^2^ = 12.58; *p* < 0.001). They also more frequently used the handheld fan in COPD (*χ*^2^ = 8.75; *p* = 0.003), fILD (*χ*^2^ = 4.85; *p* = 0.028) and LC (*χ*^2^ = 5.63; *p* = 0.018). Moreover, they were more open to refer breathless people with fILD to PC (*χ*^2^ = 5.83; *p* = 0.016), and to use pulmonary rehabilitation in COPD (*χ*^2^ = 6.41, *p* = 0.011). The subsequent comparison between knowledge of guidelines (transformed into Likert scale) and clinical practice supported these relationships (Fig. 2). There was no clear relationship between knowledge of guidelines and treatment with benzodiazepines and antidepressants. However, for fILD antidepressants were used more frequently by respondents who reported they had read guidelines or looked at them briefly (*χ*^2^ = 6.25; *p* = 0.044).

## Discussion

This is the first multinational survey to compare the experiences and attitudes of RM and PC physicians in the management of chronic refractory breathlessness in advanced COPD, fILD and LC. The responses reveal significant differences in choice of management strategy by specialty and by diagnosis. Our findings suggest a relationship between knowledge of clinical guidelines and routine use of the handheld fan, opioids, and physical activity/pulmonary rehabilitation in COPD, all of these being interventions for which there is a strong evidence base [11, 12, 21]. Although we cannot interpret this association as causality, it is possible that physicians who read guidelines are more likely to implement the evidence-based interventions recommended therein.

Our survey, in common with findings of previous national and international surveys [14, 15], found that the majority of RM physicians focus on pulmonary rehabilitation and physical activity as treatment options for breathlessness in COPD, whereas PC physicians prioritize drug treatment, most commonly an opioid. Promotion of physical activity and pulmonary rehabilitation by RM physicians is in line with international COPD practice guidelines informed by a robust evidence base [21], and indeed pulmonary rehabilitation was more likely to be favoured by respondents who reported knowledge of clinical guidelines. RM physicians prioritised drug treatment only in the LC case, and almost one fifth stated that they would never initiate opioids in severe COPD or fILD. This reluctance to initiate opioids is somewhat at odds with moderate evidence in support of low-dose opioids for chronic breathlessness in malignant and non-malignant disease [12, 13], and recommendations in current prominent international and national practice guidelines [22–24]. Again, physicians who read guidelines either carefully or looked at them briefly more often reported routine use of opioids to relieve chronic breathlessness in severe COPD. As in prior surveys [14, 15, 17, 18], RM physicians identified risk of respiratory depression to be the principal barrier to opioid prescription.

Over one-third of both PC and RM physicians reported they would routinely initiate benzodiazepines for breathlessness in LC. A quarter of PC physicians and 12% of RM physicians also reported they would routinely recommend benzodiazepines for breathlessness in fILD and 33% of PC physicians would routinely recommend also for COPD. Trials for benzodiazepines have failed to provide evidence of benefit [19], therefore, it is concerning that our survey indicates continued routine use despite this lack of evidence. In COPD, use of benzodiazepines is contrary to international practice guidelines [22]. Recent evidence suggesting an association between higher-dose benzodiazepines and mortality in fILD should also prompt caution [25]. However, guidance documents on the use of benzodiazepines for breathlessness are in some cases conflicting and at odds with current evidence. The Global Initiative for Chronic Obstructive Lung Disease (GOLD) stipulates that there is no evidence for benzodiazepine use for breathlessness in COPD [22], but benzodiazepines remain a recommended treatment option for breathlessness at rest, alone or in combination with opioids, in UK National Institute for Health and Care Excellence (NICE) Guidelines on Idiopathic Pulmonary Fibrosis [26]. Prominent PC guidelines for advanced cancer also recommend benzodiazepines for intractable breathlessness, but only in combination with opioids, and where there is co-existent anxiety. A presence of anxiety was not included in any of the case vignettes presented in our survey, however it is possible that co-existent anxiety was considered by respondents when indicating their use of benzodiazepines [27]. Without consistent, evidence-based guidelines, it is difficult, even for physicians who read all available guidelines carefully, to implement best practice across all disease settings.

Consistent with current good practice—given the lack of evidence to support their use—routine use of antidepressants in the management of breathlessness were infrequent in our survey in both RM and PC physicians, and across conditions. Moreover, one third of RM and almost half of PC physicians stated they would not use antidepressants solely for the management of breathlessness. We found no clear relationship between knowledge of guidelines and use of antidepressants. Despite promising effects in pilot work [28–30], the antidepressant sertraline did not provide any benefit over placebo in the symptomatic relief of breathlessness in a recent double-blind randomized trial [16]. The NaSSA mirtazapine is a promising candidate for the palliation of breathlessness, with definitive randomized controlled trials awaited to determine its efficacy and safety in advanced disease [31–33].

Although PC physicians prioritised drug treatments, our results show they are also open to a wider range of non-pharmacological, self-help interventions including breathing techniques, anxiety management and the handheld fan compared to RM physicians. This is in keeping with guidance for the management of breathlessness in advanced disease which recommend a combination of non-pharmacological and pharmacological interventions, with an emphasis on self-help strategies [11, 34, 35]. The handheld fan, facial cooling, mobility aids and neuromuscular electrical stimulation are all evidence-based non-pharmacological interventions for chronic refractory breathlessness [11]. Notably, more than half of RM physicians reported never recommending use of a handheld fan in breathless patients irrespective of diagnosis. This is perhaps surprising given the evidence supporting a role for this simple portable intervention, which has no major side-effects, in reducing recovery time from episodic breathlessness [36]. Better knowledge of clinical practice guidelines was related to more frequent use of the handheld fan in breathless people across conditions. It is possible that non-pharmacological treatment choices are influenced by the settings in which physicians predominantly work as well local availability of services.

Encouragingly, most RM physicians reported they would refer breathless patients with non-malignant lung disease to PC services. Randomised trial and meta-analysis evidence indicates that integrated, holistic breathlessness services reduce patient distress and may improve psychological outcomes [35, 37–41]. The COVID-19 pandemic, has highlighted, more than ever, the importance of integrated care. The breathlessness management section of the COVID-19 rapid guideline for managing symptoms (including at the end of life) in the community, is a great example of the benefits of cross-specialty working and knowledge exchange [42].

Although our findings suggest a relationship between knowledge of clinical guidelines and routine use of a breathlessness score, use of a breathlessness score is by no means adopted as routine practice, especially among PC physicians. Scoring and documentation of breathlessness is an important part of clinical assessment providing insights into disease burden and prognosis not captured by lung function alone [43]. In our survey, 62% of RM physicians reported using a breathlessness score in routine clinical practice, compared to only 13% of PC physicians. One potential explanation for this difference is that use of the mMRC Dyspnoea Score and/or COPD Assessment Test score is advocated by GOLD in the refined “ABCD” assessment of COPD disease severity [22], for which there is no counterpart in PC medicine. It is also possible that PC physicians assess breathlessness as part of a holistic assessment using tools such as the Integrated Palliative Care Outcome Scale [44] or Edmonton Symptom Assessment Scale [45] rather than a breathlessness score.

### Strengths and limitations

This multinational survey was the first to explore the management practices of physicians in RM and PC across a range of chronic advanced lung diseases. Particular attention was paid to non-malignant diseases, including ILD for which the evidence base for symptom management is poor and PC expertise remains conspicuously inaccessible [2]. However, our fILD case considered a patient with idiopathic pulmonary fibrosis specifically, thus the findings may not be generalizable to non-fibrotic ILD. The survey was distributed via society newsletter mailing lists, however as these lists contain non-physician members (e.g. researchers, students, academics, allied health professionals) it is difficult to calculate the exact response rate for our survey or consider the characteristics of non-responders. Responder bias therefore needs to be considered. Unfortunately, there was a high number of incomplete questionnaires (n = 250) which could not be included in the analysis as responses allowing identification of respondents’ specialty were missing. However, the analysed sample size is comparable to other recent surveys.(14, 15) The majority of responses were from the UK and therefore our findings may not represent practice across different healthcare systems. Finally, self-reported knowledge and attitudes to management of case vignettes may not reflect actual clinical practice.

## Conclusions

This survey of RM and PC physicians reveals substantial differences in the approach to clinical management of chronic refractory breathlessness between specialties, and between malignant and non-malignant lung disease. The findings suggest that knowledge of clinical practice guidelines influences evidence-based treatment choices, but there was also evidence of deviation from current recommendations, particularly related to the use of benzodiazepines. There is a need for randomized clinical trials of new drug treatments, including antidepressants, to clarify clinical efficacy and reduce ambiguities in current practice recommendations. Most RM physicians welcomed the opportunity for shared care with PC colleagues. Together, these findings emphasise the need for improved dissemination and uptake of joint, evidence-based clinical practice guidelines, developed and ratified by major palliative care and respiratory societies, to reduce the significant burden of chronic refractory breathlessness in advanced respiratory disease.

## Supplementary Information

**Additional file 1.** Copy of survey questions.

## Data Availability

The datasets used and/or analysed during the current study are available from the corresponding author on reasonable request.

## Abbreviations

BTS: British Thoracic Society
CI: Confidence Interval
COPD: Chronic Obstructive Pulmonary Disease
EAPC: European Association of Palliative Care
ERS: European Respiratory Society
fILD: Fibrotic Interstitial Lung Disease
GOLD: The Global Initiative for Chronic Obstructive Lung Disease
ILD: Interstitial Lung Disease
IPF: Idiopathic Pulmonary Fibrosis
LC: Lung Cancer
mMRC: Modified Medical Research Council Dyspnea Scale
NaSSA: Noradrenergic and Specific Serotonergic Antidepressant
NICE: National Institute for Health and Care Excellence
OR: Odds Ratio
RM: Respiratory Medicine
PC: Palliative Care
PCF: Palliative Care Formulary
SSRI: Selective Serotonin Reuptake Inhibitor
UK: United Kingdom
USA: Unites States of America
