# Supplementary material for: Do guidelines influence breathlessness management in advanced lung diseases? A multinational survey of respiratory medicine and palliative care physicians
Source: BMC Pulm Med. 2022 Jan 19;22:41. doi: 10.1186/s12890-022-01835-0 (PMC8768441; doi:10.1186/s12890-022-01835-0)
Supplement: Supplementary file 1 — Additional file 1. Copy of survey questions. [file 12890_2022_1835_MOESM1_ESM.docx]

### Additional File 1: Copy of survey questions

**Physicians’ attitudes and experiences of managing breathlessness in palliative, respiratory and end of life care**

**As part of the EU funded BETTER-B project, we are inviting physicians to complete this voluntary, online survey which should take no more than 15 minutes.

The survey focuses on the management of chronic breathlessness for patients with severe chronic obstructive pulmonary disease (COPD), interstitial lung disease (ILD) and lung cancer i.e. their breathlessness persists despite optimal treatment of the underlying causes.

We are particularly interested in your views regarding non-drug and drug treatments for chronic breathlessness. The survey is designed to explore individual physicians’ beliefs and experiences, and there are no right or wrong answers.

In compliance with the General Data Protection Regulation 2016/679, the information you provide will be anonymous and confidential. Your information will be stored on a password-protected university network and will not be passed on to anyone outside of the research team.

If you wish to receive a summary of the results, you may provide your email address at the end of the survey. These details will be kept separately from the survey itself to retain anonymity.

This study has been reviewed and given ethical approval by the King’s College London (UK) Research Ethics Committee (MRA-18/19-11108). If you would like any further information please contact the project manager:

Dr Nilay Hepgul
better-b@kcl.ac.uk

By starting the survey, you are indicating your consent to participate.

Thank you**

**Do you know of any local, national or international guidelines/recommendations on palliative care for non-malignant lung diseases?**

|  | Yes, I know of them and have read them carefully |
| --- | --- |
|  | Yes, I know of them but have only looked at them briefly |
|  | Yes, I know of them but have not read them |
|  | I know that no such guidelines/recommendations exist |
|  | I’m not sure if such guidelines/recommendations exist or not |

If you have answered yes above, please list them:

|  |
| --- |

**Do you routinely use a breathlessness score in clinical practice (e.g. Borg, modified MRC, other) to quantify your patients' breathlessness?**

|  | Yes, I routinely use a breathlessness score |
| --- | --- |
|  | Yes, I sometimes use a breathlessness score |
|  | No, I never use a breathlessness score |
|  | No, I don’t know any breathlessness scores |

**MARTIN**

**Martin is a 75 year old man who is an ex-smoker with very severe COPD (i.e. GOLD grade 4, group D), treated optimally according to the GOLD guidelines (2019). He has been prescribed home nebulisers and long-term oxygen therapy (LTOT) for the past 8 months. His FEV1 is 0.8L (27% predicted), SpO2 is 91% on 2L/min O2, and he is currently normocapnic.**

**Last winter Martin needed non-invasive ventilation for acute hypercapnic respiratory failure, but he has never been intubated. Martin is now breathless at rest, and severely deconditioned. His breathlessness becomes intolerable after just a few steps, even using ambulatory oxygen, and he can no longer walk across the room unaided.**

**On average how many patients per year do you see who are similar to the case described?**

|  | None |
| --- | --- |
|  | 1 - 10 per year |
|  | 11 - 50 per year |
|  | 51 - 100 per year |
|  | 101 - 150 per year |
|  | >150 per year |

**How often do you recommend non-pharmacological treatment specifically for chronic breathlessness for a patient with very severe COPD on LTOT similar to Martin?**

|  | Never (0% of similar cases) | Rarely (about 25% of similar case) | Sometimes (about 50% of similar cases) | Often (about 75% of similar cases) | Always (100% of similar cases) |
| --- | --- | --- | --- | --- | --- |
| Pulmonary rehabilitation |  |  |  |  |  |
| Physical activity |  |  |  |  |  |
| Electric handheld fan |  |  |  |  |  |
| Breathing techniques |  |  |  |  |  |
| Respiratory muscle training |  |  |  |  |  |
| Body positioning to relieve breathlessness |  |  |  |  |  |
| Walking aids |  |  |  |  |  |
| Meditative interventions |  |  |  |  |  |
| Cognitive- emotional interventions |  |  |  |  |  |

Other non-pharmacological treatment (please specify what and how often):

|  |
| --- |

**How often would you start the following pharmacological treatments for a patient with very severe COPD on LTOT similar to Martin, who now has chronic breathlessness at rest despite optimal treatment of their COPD?**

|  | Never (0% of similar cases) | Rarely (about 25% of similar cases) | Sometimes (about 50% of similar cases) | Often (about 75% of similar cases) | Always (100% of similar cases) |
| --- | --- | --- | --- | --- | --- |
| Opioids |  |  |  |  |  |
| Benzodiazepines |  |  |  |  |  |
| Antidepressants |  |  |  |  |  |

Other pharmacological treatments (please specify what and how often):

|  |
| --- |

**If you use opioid medication to treat chronic breathlessness in a patient with very severe COPD like Martin, what would be your starting dose (please choose only one option that you use most often)?**

|  | Oral dihydrocodeine regularly (every 4-6 hours) |
| --- | --- |
|  | Short-acting oral morphine only as required (e.g. morphine 2.5-5mg every 4-6 hours as required) |
|  | Short-acting oral morphine regularly (e.g. morphine 2.5-5mg every 4-6 hours) |
|  | Long-acting oral morphine (e.g. 10-20mg/24 hours) |
|  | Subcutaneous morphine injection (e.g. morphine 2.5-5mg, or less) as required |
|  | Subcutaneous morphine injection regularly or as a continuous infusion |
|  | Other (please specify opioid, starting dose, and route of administration):   \|  \| \| --- \| |

**Please indicate why you do not, or only rarely, prescribe opioids to a patient with very severe COPD, on LTOT who now has chronic breathlessness at rest, similar to Martin (select all that apply)**

|  | I do not have enough knowledge or experience prescribing opioids to COPD patients with chronic breathlessness |
| --- | --- |
|  | I am not comfortable prescribing potentially addictive drugs |
|  | Opioids are not effective for the treatment of chronic breathlessness |
|  | I am not aware of any guidelines suggesting opioids are useful for treating chronic breathlessness |
|  | Opioids may have unpleasant side-effects (nausea, vomiting, constipation, drowsiness) |
|  | Opioids may cause respiratory depression |
|  | Opioids are contraindicated in patients with hypercapnoea |
|  | Opioids only have a role in breathless patients who are in the terminal phase of their illness |
|  | Opioids should be initiated by a Palliative Medicine specialist |
|  | Other (please specify):   \|  \| \| --- \| |

**If you use a benzodiazepine to treat chronic breathlessness in a patient with very severe COPD like Martin, what kind of treatment do you choose? (please choose the option that you use most often)**

|  | Long-acting benzodiazepine orally (e.g. diazepam) regularly |
| --- | --- |
|  | Long-acting benzodiazepine orally (e.g. diazepam) only as required |
|  | Intermediate-acting benzodiazepine orally (e.g. lorazepam) regularly |
|  | Intermediate-acting benzodiazepine orally (e.g. lorazepam) only as required |
|  | Short-acting benzodiazepine subcutaneously (e.g. midazolam) only as required |
|  | Short-acting benzodiazepine subcutaneous (e.g. midazolam) regularly |
|  | Other (please specify benzodiazepine, starting dose, and route of administration):   \|  \| \| --- \| |

**If you use antidepressants to treat chronic breathlessness in a patient with very severe COPD like Martin, what kind of treatment do you choose? (please choose the option that you use most often)**

|  | An SSRI e.g. sertraline, paroxetine, fluoxetine, citalopram |
| --- | --- |
|  | A NaSSA e.g. mirtazapine |
|  | A tricyclic antidepressant e.g. amitriptyline |
|  | A SNRI e.g. venlafaxine |
|  | I do not use antidepressants to treat chronic breathlessness only |
|  | Other (please specify):   \|  \| \| --- \| |

**Indicate your main reason for prescribing an antidepressant in a patient with very severe COPD like Martin?**

|  | To treat chronic breathlessness |
| --- | --- |
|  | To treat comorbid depression |
|  | To treat comorbid anxiety |
|  | To treat insomnia |
|  | Other (please specify):   \|  \| \| --- \| |

**Which management option would you prioritise, as the most important first step, to improve chronic breathlessness at rest in a patient with very severe COPD, on LTOT like Martin? (choose one option)**

|  | Drug treatment for breathlessness |
| --- | --- |
|  | Re-assess oxygen prescription |
|  | Non-pharmacological, non-exercise intervention for breathlessness specifically, e.g. electric fan |
|  | Exercise training / rehabilitation |
|  | Psychological assessment to explore co-existing anxiety and/or depression |
|  | Other (please specify):   \|  \| \| --- \| |

**Would you refer patients with very severe COPD like Martin to a Palliative Care service?**

|  | Yes - for advice about palliation of breathlessness only |
| --- | --- |
|  | Yes - to provide ongoing palliation of breathlessness and other symptoms along with psychosocial and spiritual support (including community/home palliative care) |
|  | I don't know |
|  | No |
|  | Not applicable (I am a Palliative Care Physician) |
|  | Other (please specify):   \|  \| \| --- \| |

**TONY**

**Tony is a 61 year old man with severe idiopathic pulmonary fibrosis (IPF) still on antifibrotic treatment. His FVC is 50% predicted, DLCO 32% predicted and SpO2 is currently 92% at rest. He has no history of heart disease, anxiety or depression. Tony identifies worsening breathlessness as his main concern. Although not breathless at rest, he now struggles with breathlessness when walking, bathing, or speaking. Tony had been prescribed oxygen during a recent hospital admission but did not find that it helped his breathlessness.**

**On average how many patients with with severe interstitial lung disease (ILD), similar to the case described, do you see per year?**

|  | None |
| --- | --- |
|  | 1 - 5 per year |
|  | 6 - 10 per year |
|  | 11 - 20 per year |
|  | >20 per year |

**How often do you recommend non-pharmacological treatment specifically for a patient with severe interstitial lung disease (ILD) who presents with chronic breathlessness on minimal exertion, similar to Tony?**

|  | Never (0% of similar cases) | Rarely (about 25% of similar case) | Sometimes (about 50% of similar cases) | Often (about 75% of similar cases) | Always (100% of similar cases) |
| --- | --- | --- | --- | --- | --- |
| Pulmonary rehabilitation |  |  |  |  |  |
| Physical activity |  |  |  |  |  |
| Electric handheld fan |  |  |  |  |  |
| Breathing techniques |  |  |  |  |  |
| Respiratory muscle training |  |  |  |  |  |
| Body positioning to relieve breathlessness |  |  |  |  |  |
| Walking aids |  |  |  |  |  |
| Meditative interventions |  |  |  |  |  |
| Cognitive- emotional interventions |  |  |  |  |  |

Other non-pharmacological treatment (please specify what and how often):

|  |
| --- |

**How often would you start the following pharmacological treatments for a patient with severe ILD who presents with chronic breathlessness on minimal exertion, similar to Tony?**

|  | Never (0% of similar cases) | Rarely (about 25% of similar cases) | Sometimes (about 50% of similar cases) | Often (about 75% of similar cases) | Always (100% of similar cases) |
| --- | --- | --- | --- | --- | --- |
| Opioids |  |  |  |  |  |
| Benzodiazepines |  |  |  |  |  |
| Antidepressants |  |  |  |  |  |

Other pharmacological treatments (please specify what and how often):

|  |
| --- |

**If you use opioid medication for a patient with severe ILD who presents with chronic breathlessness on minimal exertion, similar to Tony, what would be your starting dose? (please choose the option that you use most often)**

|  | Oral dihydrocodeine regularly (every 4-6 hours) |
| --- | --- |
|  | Short-acting oral morphine only as required (e.g. morphine 2.5-5mg every 4-6 hours as required) |
|  | Short-acting oral morphine regularly (e.g. morphine 2.5-5mg every 4-6 hours) |
|  | Long-acting oral morphine (e.g. 10-20mg/24 hours) |
|  | Subcutaneous morphine injection (e.g. morphine 2.5-5mg, or less) as required |
|  | Subcutaneous morphine injection regularly or as a continuous infusion |
|  | Other (please specify opioid, starting dose, and route of administration):   \|  \| \| --- \| |

**Please indicate why you do not, or only rarely, prescribe opioids to a patient with severe ILD who presents with chronic breathlessness on minimal exertion, similar to Tony (select all that apply).**

|  | I do not have enough knowledge or experience prescribing opioids to ILD patients with chronic breathlessness |
| --- | --- |
|  | I am not comfortable prescribing potentially addictive drugs |
|  | Opioids are not effective for the treatment of chronic breathlessness |
|  | I am not aware of any guidelines suggesting opioids are useful for treating chronic breathlessness |
|  | Opioids may have unpleasant side-effects (nausea, vomiting, constipation, drowsiness) |
|  | Opioids may cause respiratory depression |
|  | Opioids are contraindicated in patients with hypercapnoea |
|  | Opioids only have a role in breathless patients who are in the terminal phase of their illness |
|  | Opioids should be initiated by a Palliative Medicine specialist |
|  | Other (please specify):   \|  \| \| --- \| |

**If you use a benzodiazepine to treat breathlessness in a patient with severe ILD who presents with chronic breathlessness on minimal exertion, similar to Tony, what kind of treatment do you choose? (please choose the option that you use most often)**

|  | Long-acting benzodiazepine orally (e.g. diazepam) regularly |
| --- | --- |
|  | Long-acting benzodiazepine orally (e.g. diazepam) only as required |
|  | Intermediate-acting benzodiazepine orally (e.g. lorazepam) regularly |
|  | Intermediate-acting benzodiazepine orally (e.g. lorazepam) only as required |
|  | Short-acting benzodiazepine subcutaneously (e.g. midazolam) only as required |
|  | Short-acting benzodiazepine subcutaneous (e.g. midazolam) regularly |
|  | Other (please specify benzodiazepine, starting dose, and route of administration):   \|  \| \| --- \| |

**If you use antidepressants to treat breathlessness in a patient with severe ILD who presents with chronic breathlessness on minimal exertion, similar to Tony, what kind of treatment do you choose? (please choose the option that you use most often)**

|  | An SSRI e.g. sertraline, paroxetine, fluoxetine, citalopram |
| --- | --- |
|  | A NaSSA e.g. mirtazapine |
|  | A tricyclic antidepressant e.g. amitriptyline |
|  | A SNRI e.g. venlafaxine |
|  | I do not use antidepressants to treat chronic breathlessness only |
|  | Other (please specify):   \|  \| \| --- \| |

**Indicate your main reason for prescribing an antidepressant in a patient with severe ILD like Martin?**

|  | To treat chronic breathlessness |
| --- | --- |
|  | To treat comorbid depression |
|  | To treat comorbid anxiety |
|  | To treat insomnia |
|  | Other (please specify):   \|  \| \| --- \| |

**Which management option would you prioritise, as the most important first step, to improve a patient with severe ILD who presents with chronic breathlessness on minimal exertion, similar to Tony? (choose one option)**

|  | Drug treatment for breathlessness |
| --- | --- |
|  | Re-assess oxygen prescription |
|  | Non-pharmacological, non-exercise intervention for breathlessness specifically, e.g. electric fan |
|  | Exercise training / rehabilitation |
|  | Psychological assessment to explore co-existing anxiety and/or depression |
|  | Other (please specify):   \|  \| \| --- \| |

**Would you refer patients with severe ILD like Tony to a Palliative Care service?**

|  | Yes - for advice about palliation of breathlessness only |
| --- | --- |
|  | Yes - to provide ongoing palliation of breathlessness and other symptoms along with psychosocial and spiritual support (including community/home palliative care) |
|  | I don't know |
|  | No |
|  | Not applicable (I am a Palliative Care Physician) |
|  | Other (please specify):   \|  \| \| --- \| |

**HELENA**

**Helena is an 82 year old woman with advanced (stage 4) lung cancer being cared for at home. Her SpO2 is 96% breathing room air. She has a poor performance status (ECOG 2-3). She is breathless at rest and she has found this increasingly distressing over the past 2 weeks. She has no history of heart disease, anxiety or depression.**

**On average how many patients per year do you see who are similar to the case described?**

|  | None |
| --- | --- |
|  | 1 - 10 per year |
|  | 11 - 50 per year |
|  | 51 - 100 per year |
|  | 101 - 150 per year |
|  | >150 per year |

**How often do you recommend non-pharmacological treatment specifically for chronic breathlessness at rest in a patient with advanced lung cancer, similar to Helena?**

|  | Never (0% of similar cases) | Rarely (about 25% of similar case) | Sometimes (about 50% of similar cases) | Often (about 75% of similar cases) | Always (100% of similar cases) |
| --- | --- | --- | --- | --- | --- |
| Pulmonary rehabilitation |  |  |  |  |  |
| Physical activity |  |  |  |  |  |
| Electric handheld fan |  |  |  |  |  |
| Breathing techniques |  |  |  |  |  |
| Respiratory muscle training |  |  |  |  |  |
| Body positioning to relieve breathlessness |  |  |  |  |  |
| Walking aids |  |  |  |  |  |
| Meditative interventions |  |  |  |  |  |
| Cognitive- emotional interventions |  |  |  |  |  |

Other non-pharmacological treatment (please specify what and how often):

|  |
| --- |

**How often would you start the following pharmacological treatments for chronic breathlessness at rest in a patient with advanced lung cancer, similar to Helena?**

|  | Never (0% of similar cases) | Rarely (about 25% of similar cases) | Sometimes (about 50% of similar cases) | Often (about 75% of similar cases) | Always (100% of similar cases) |
| --- | --- | --- | --- | --- | --- |
| Opioids |  |  |  |  |  |
| Benzodiazepines |  |  |  |  |  |
| Antidepressants |  |  |  |  |  |

Other pharmacological treatments (please specify what and how often):

|  |
| --- |

**If you use opioid medication to treat chronic breathlessness in a patient with advanced lung cancer, similar to Helena, what would be your starting dose (please choose only one option that you use most often)?**

|  | Oral dihydrocodeine regularly (every 4-6 hours) |
| --- | --- |
|  | Short-acting oral morphine only as required (e.g. morphine 2.5-5mg every 4-6 hours as required) |
|  | Short-acting oral morphine regularly (e.g. morphine 2.5-5mg every 4-6 hours) |
|  | Long-acting oral morphine (e.g. 10-20mg/24 hours) |
|  | Subcutaneous morphine injection (e.g. morphine 2.5-5mg, or less) as required |
|  | Subcutaneous morphine injection regularly or as a continuous infusion |
|  | Other (please specify opioid, starting dose, and route of administration):   \|  \| \| --- \| |

**Please indicate why you do not, or only rarely, prescribe opioids to treat chronic breathlessness at rest in a patient with advanced lung cancer, similar to Helena. (select all that apply)**

|  | I do not have enough knowledge or experience prescribing opioids to lung cancer patients with chronic breathlessness |
| --- | --- |
|  | I am not comfortable prescribing potentially addictive drugs |
|  | Opioids are not effective for the treatment of chronic breathlessness |
|  | I am not aware of any guidelines suggesting opioids are useful for treating chronic breathlessness |
|  | Opioids may have unpleasant side-effects (nausea, vomiting, constipation, drowsiness) |
|  | Opioids may cause respiratory depression |
|  | Opioids are contraindicated in patients with hypercapnoea |
|  | Opioids only have a role in breathless patients who are in the terminal phase of their illness |
|  | Opioids should be initiated by a Palliative Medicine specialist |
|  | Other (please specify):   \|  \| \| --- \| |

**If you use a benzodiazepine to treat chronic breathlessness at rest in a patient with advanced lung cancer, similar to Helena, what kind of treatment do you choose? (please choose the option that you use most often)**

|  | Long-acting benzodiazepine orally (e.g. diazepam) regularly |
| --- | --- |
|  | Long-acting benzodiazepine orally (e.g. diazepam) only as required |
|  | Intermediate-acting benzodiazepine orally (e.g. lorazepam) regularly |
|  | Intermediate-acting benzodiazepine orally (e.g. lorazepam) only as required |
|  | Short-acting benzodiazepine subcutaneously (e.g. midazolam) only as required |
|  | Short-acting benzodiazepine subcutaneous (e.g. midazolam) regularly |
|  | Other (please specify benzodiazepine, starting dose, and route of administration):   \|  \| \| --- \| |

**If you use antidepressants to treat chronic breathlessness at rest in a patient with advanced lung cancer, similar to Helena, what kind of treatment do you choose? (please choose the option that you use most often)**

|  | An SSRI e.g. sertraline, paroxetine, fluoxetine, citalopram |
| --- | --- |
|  | A NaSSA e.g. mirtazapine |
|  | A tricyclic antidepressant e.g. amitriptyline |
|  | A SNRI e.g. venlafaxine |
|  | I do not use antidepressants to treat chronic breathlessness only |
|  | Other (please specify):   \|  \| \| --- \| |

**Indicate your main reason for prescribing an antidepressant in a patient with advanced lung cancer, like Helena?**

|  | To treat chronic breathlessness |
| --- | --- |
|  | To treat comorbid depression |
|  | To treat comorbid anxiety |
|  | To treat insomnia |
|  | Other (please specify):   \|  \| \| --- \| |

**Which management option would you prioritise, as the most important first step, to improve chronic breathlessness at rest in a patient with advanced lung cancer, similar to Helena? (choose one option)**

|  | Drug treatment for breathlessness |
| --- | --- |
|  | Re-assess oxygen prescription |
|  | Non-pharmacological, non-exercise intervention for breathlessness specifically, e.g. electric fan |
|  | Exercise training / rehabilitation |
|  | Psychological assessment to explore co-existing anxiety and/or depression |
|  | Other (please specify):   \|  \| \| --- \| |

**Would you refer advanced lung cancer patients with chronic breathlessness like Helena to a Palliative Care service?**

|  | Yes - for advice about palliation of breathlessness only |
| --- | --- |
|  | Yes - to provide ongoing palliation of breathlessness and other symptoms along with psychosocial and spiritual support (including community/home palliative care) |
|  | I don't know |
|  | No |
|  | Not applicable (I am a Palliative Care Physician) |
|  | Other (please specify):   \|  \| \| --- \| |

**What is your country of practice?**

| Choose from drop-down menu |
| --- |

Other (please specify):

|  |
| --- |

**What is your specialty (Please select all that apply)**

|  | Pulmonology/respiratory medicine |
| --- | --- |
|  | Palliative medicine |
|  | Other (please specify):   \|  \| \| --- \| |

**What is your current grade?**

|  | Consultant/specialist |
| --- | --- |
|  | Doctor on a specialist training program |
|  | Other (please specify):   \|  \| \| --- \| |

**In which settings do you practice? (Please select all that apply)**

|  | Hospital inpatient |
| --- | --- |
|  | Outpatient |
|  | Home care |

|  | Private practice |
| --- | --- |
|  | Hospice/palliative care unit |
|  | Other (please specify):   \|  \| \| --- \| |

**How many years have you worked in your specialty? (Please include also years in specialist training)**

|  | Less than 5 |
| --- | --- |
|  | 6-10 |
|  | 11-20 |

|  | 21-30 |
| --- | --- |
|  | 31-40 |
|  | More than 40 |

**What is your age?**

|  | 25-30 |
| --- | --- |
|  | 31-35 |
|  | 36-40 |
|  | 41-45 |
|  | 46-50 |

|  | 51-55 |
| --- | --- |
|  | 56-60 |
|  | 61-65 |
|  | Over 65 |
|  | Prefer not to say |
